# Immunoprofiling of 4-1BB Expression Predicts Outcome in Chronic Lymphocytic Leukemia (CLL)

**DOI:** 10.3390/diagnostics11112041

**Published:** 2021-11-04

**Authors:** Kübra Kaban, Sarah M. Greiner, Samuel Holzmayer, Claudia Tandler, Sophie Meyer, Clemens Hinterleitner, Helmut R. Salih, Melanie Märklin, Jonas S. Heitmann

**Affiliations:** 1 Clinical Collaboration Unit Translational Immunology, German Cancer Consortium (DKTK), Department of Internal Medicine, University Hospital Tübingen, 72076 Tübingen, Germany; kuebra.kaban@gmail.com (K.K.); sarah.maria.greiner@med.uni-tuebingen.de (S.M.G.); samuel.holzmayer@med.uni-tuebingen.de (S.H.); claudia.tandler@med.uni-tuebingen.de (C.T.); sophie.meyer@student.uni-tuebingen.de (S.M.); helmut.salih@med.uni-tuebingen.de (H.R.S.); jonas.heitmann@med.uni-tuebingen.de (J.S.H.); 2 DFG Cluster of Excellence 2180 ‘Image-guided and Functional Instructed Tumor Therapy’ (iFIT), University of Tübingen, 72076 Tübingen, Germany; clemens.hinterleitner@med.uni-tuebingen.de; 3 Department of Medical Oncology and Pulmonology, University Hospital Tübingen, 72076 Tübingen, Germany

**Keywords:** CLL, 4-1BB/4-1BBL, GITR/GITRL, survival

## Abstract

Recent success of novel therapies has improved treatment of chronic lymphocytic leukemia (CLL) patients, but most of them still require several treatment regimes. To improve treatment choice, prognostic markers suitable for prediction of disease outcome are required. Several molecular/genetic markers have been established, but accessibility for the entirety of all patients is limited. We here evaluated the relevance of GITR/4-1BB as well as their ligands for the prognosis of CLL patients. Surface expression of GITR/GITRL and 4-1BB/4-1BBL was correlated with established prognostic markers. Next, we separated our patient population according to GITR/GITRL and 4-1BB/4-1BBL expression in groups with high/low expression levels and performed Kaplan-Meier analyses. Interestingly, no correlation was observed with the defined prognostic markers. Whereas no significant difference between high and low expression of GITR, GITRL and 4-1BBL was observed, high 4-1BB levels on leukemic cells were associated with significantly shorter survival. Thereby we identify 4-1BB as prognostic marker for CLL.

## 1. Introduction

Treatment options of chronic lymphocytic leukemia (CLL) patients have greatly improved over recent years [1,2]. An increasing understanding of disease pathophysiology led to the development of novel small molecules with tremendous clinical efficiency. Accordingly, for example, ibrutinib, which targets Bruton’s tyrosine kinase (BTK), has been approved as first line treatment for CLL patients [3,4]. In addition to this and other small molecule inhibitors like venetoclax and idelalisib (for review see [5]), immunotherapy comprising anti-CD20 antibodies remains a central option in CLL treatment. In general, it is still a matter of debate when treatment should be initiated: directly upon diagnosis or upon progression of disease. To address this issue, several clinical trials have been initiated, e.g., the CLL12 trial treating Binet A patients [6,7,8], and several prognostic markers have been implemented to guide treatment decision in the last decades [9,10]. In addition to age, molecular and cyto-/genetic profiling have identified the mutational status of the immunoglobulin heavy-chain variable region (IGHV), TP53 mutation and chromosomal abnormalities such as del13q14, del11q22-23, del17p12, and trisomy 12 as relevant prognostic markers [11,12]. Even earlier, immunophenotyping of CLL cells identified ZAP70 and CD38 expression as predictors of disease course [13]. However, even though both are rapidly and more frequently available, the appreciation for both has decreased with the discovery of the aforementioned molecular markers, of which both IGHV and TP53 mutation status also guide treatment decision in terms of applied therapy [9].

Glucocorticoid-induced TNFR-related protein (GITR, TNFRSF18) and its ligand (GITRL) are members of the TNF superfamily of proteins and have been shown to influence multiple physiological and pathophysiological conditions [14,15,16,17]. GITR is expressed on multiple different cell types such as T cells, macrophages, dendritic cells, and B cells, and its expression level is regulated by activation [18,19,20]. Its counterpart GITRL is highly expressed on endothelial cells and activated antigen-presenting cells (APCs) like B cells [21], but also on tumor cells [22,23,24,25]. Whereas the function of GITR/GITRL has been initially investigated with a focus on regulatory T cells [26,27], their functional role in malignant cells and anti-tumor immunity has more recently attracted considerable interest.

Likewise, members of the TNF superfamily, 4-1BB (CD137, TNFRSF9) and its ligand 4-1BBL are membrane glycoproteins expressed by a wide variety of cells [28]. 4-1BB is predominantly expressed on activated CD4^+^ and CD8^+^ T cells. However, expression has also been reported for activated NK cells, monocytes, dendritic cells, B cells, chondrocytes, mast cells, and malignant cells [28,29]. Its counterpart 4-1BBL is expressed predominantly on APCs, but also on malignant cells [30]. In recent years, that the ability of 4-1BB to co-stimulate survival and effector function of CD4^+^ and particularly CD8^+^ T cells received the most interest [31,32]. For both GITR/GITRL and 4-1BB/4-1BBL, bidirectional receptor-ligand interaction also affects multiple other healthy and malignant cell types, including CLL [33], but association of expression with survival of CLL patients has so far not been studied.

In this study, we investigated whether immunophenotyping for GITR/GITRL and 4-1BB/4-1BBL expression in CLL (*n* = 73) could serve as a predictor of disease outcome.

## 2. Materials and Methods

### 2.1. Patient Samples

Peripheral blood samples of 73 patients with CLL and samples of 15 healthy donors were collected and peripheral blood mononuclear cells (PBMC) were isolated by density gradient centrifugation and used for flow cytometry. None of these analyzed samples were acquired during disease treatment. Median follow-up time for all patients was 131 months (range 13.5–200 months). Cytogenetic and molecular analyses were performed at licensed laboratories with standard methods. All procedures performed in studies involving human participants were in accordance with the ethical standards of the institutional research committee (Ethic Committee of the University of Tübingen vote 13/2007V) and with the Helsinki declaration. All participants provided written informed consent.

### 2.2. Flow Cytometry

PBMC of CLL patients and healthy donors were incubated with human IgG (Sigma-Aldrich, St. Louis, MO, USA) prior to the staining in order to minimize Fcɣ receptor binding, then washed and stained with the unconjugated GITR (clone 110416, R&D Systems, Inc., Minneapolis, MN, USA), GITRL (clone 109101, R&D Systems, Inc., Minneapolis, MN, USA), 4-1BB (clone 4B4-1, Ancell Corporation, Bayport, MN, USA), and 4-1BBL (clone C65-485, BD Pharmingen Inc., Heidelberg, Germany) mAbs or the isotype control at 10 μg/mL, followed by species-specific PE-conjugated detection antibodies (1:100). The CLL cells were then identified by staining for CD19 (clone HIB19, BD) and CD5 (clone UCHT2, BD), and dead cells were excluded based on 7-AAD (BioLegend, San Diego, CA, USA) positivity. FITC- and APC-conjugates (CD19 and CD5) were used in 1:25–1:50 dilutions, respectively. Specific fluorescence indices (SFIs) were calculated by dividing median fluorescence obtained with anti-GITR, anti-GITRL, anti-4-1BB, and anti-4 1BBL mAbs by median fluorescence obtained with the IgG1 isotype control. Positive expression was defined as SFI ≥1.5. Measurements were conducted using a BD FACSCanto™ II Flow Cytometer (BD Biosciences, Heidelberg, Germany) and data analysis was performed with FlowJo_V10.5.3 software (FlowJo LCC, BD). 

### 2.3. Statistical Analysis

Data are shown as mean +/− standard deviation (SD), boxplots including median and 25% and 75% quartiles, as well as min/max or Tukey whiskers. Continuous data were tested for distribution and individual groups were compared with the Mann-Whitney-U-test. Correlation was analyzed using Spearman’s rank correlation coefficient. Distribution of overall survival (OS) was calculated using the Kaplan-Meier method. Log-rank test or appropriate alternative were performed to compare survival between groups. For predictive estimation, we sub-grouped the data sets according to median of GITR/GITRL/4-1BB/4-1BBL SFI expression levels (low vs. high). Statistical analyses were conducted using JMP^®^ Pro and GraphPad Prism 8.4.0 software (GraphPad Software, San Diego, CA, USA). Any *p* values of <0.05 were considered statistically significant.

## 3. Results

### 3.1. Clinical Characteristics

A total of 73 CLL patients were included in the analysis. Clinical characteristics of the patients are given in Table 1. The age range was 36–80 years, with a median age of 63. Males represented 58% (*n* = 42) of the studied population. Most patients presented with Binet stage A at diagnosis (*n* = 48), 15 with stage B, and 6 with stage C. Rai stage 0 was present in 20 patients at initial diagnosis, stage I–II in 32 patients, and stage III–IV in 6 patients. IGHV mutational status was determined in 17 patients, with almost equal distribution of mutated (*n* = 10) and unmutated (*n* = 7) IGHV. Expression of other prognostic markers such as CD38 expression <20% was observed in 43 patients, 20–29% in 3 patients, and ≥30% in 15 patients. Three out of 23 patients presented with a TP53 mutation. Classical cytogenetic analysis was performed for 19 patients. Patients were grouped in three groups according to risk: favorable risk (12q trisomy, del13q), intermediate risk (normal karyotype), and poor risk (del17p, del11q). Out of tested patients 5, 4, and 10 were categorized as favorable, intermediate, and poor risk, respectively.

### 3.2. Expression Profile of GITR/GITRL and 4-1BB/4-1BBL on Peripheral CLL, NK, T Cells and Healthy Individuals

**Figure 1:**
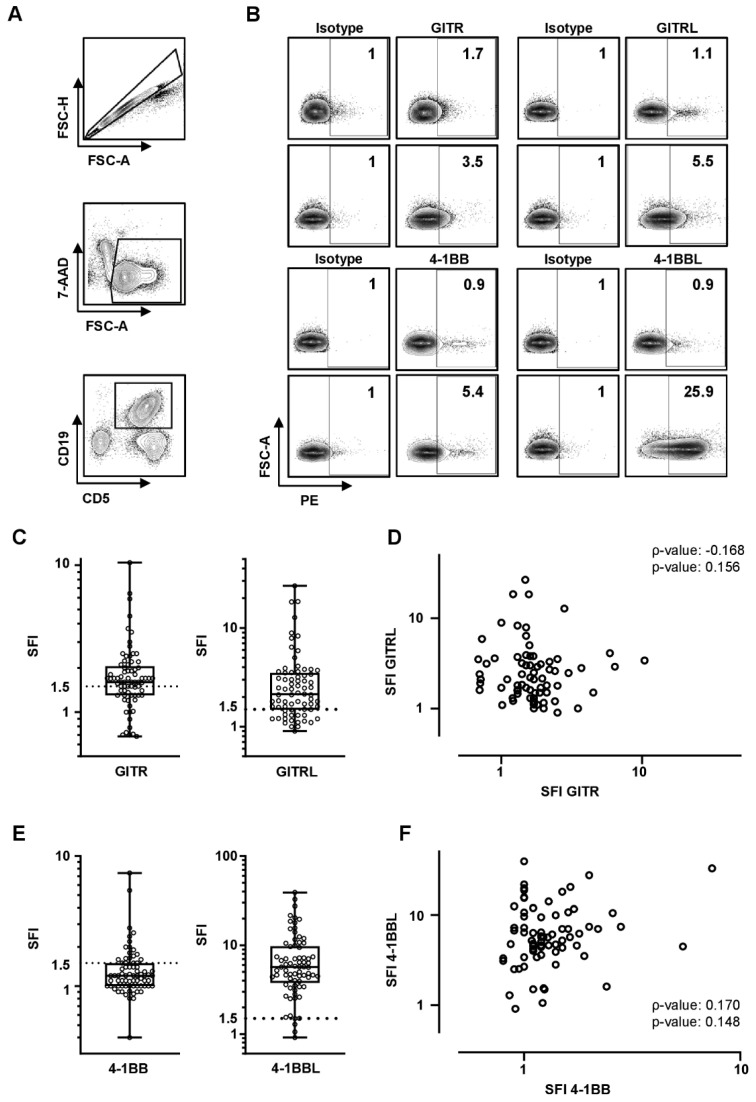
GITR/GITRL and 4-1BB/4-1BBL expression on chronic lymphocytic leukemia cells. GITR/GITRL/4-1BB/4-1BBL expression levels were analyzed on CLL cells by flow cytometry. (**A**) An exemplary gating strategy is depicted: singlets, viable (7-AAD-), CD5^+^CD19^+^ CLL cells. (**B**) Gating strategy for low and high GITR/GITRL/4-1BB/4-1BBL expression levels of exemplary CLL samples compared to isotype is depicted. SFIs are shown on the right corner of each plot. (**C**,**E**) GITR/GITRL/4-1BB/4-1BBL expression levels on peripheral CLL cells (*n* = 73) are shown as SFI levels of GITR, GITRL, 4-1BB, and 4-1BBL positive CLL samples (boxplots with min/max whiskers). The SFI ≥ 1.5 is defined as positive and is depicted by dotted line. (**D**,**F**) Correlation of GITR and GITRL, and 4-1BB and 4-1BBL SFI levels on CLL cells is shown (single values, spearman correlation (ρ)).

As an initial step, receptor and ligand expression was determined on CD5^+^CD19^+^ CLL cells by flow cytometry. An exemplary gating strategy is depicted in Figure 1A,B. Expression levels (SFI) were heterogeneously distributed among the cohort with up to SFIs of 10.4, 26.7, 7.4, and 39.0 for GITR, GITRL, 4-1BB, and 4-1BBL, respectively (Figure 1C,E). According to our predefined definition for positivity (SFI ≥ 1.5), 48, 54, 20, and 69 out of 73 patients expressed GITR, GITRL, 4-1BB, and 4-1BBL, respectively. Analysis of surface expression on CLL cells did not reveal any significant correlation for GITR and GITRL (ρ = 0.168, *p* = 0.156) or 4-1BB and 4-1BBL (ρ = 0.170, *p* = 0.148) (Figure 1D,F) between each other.

**Figure 2:**
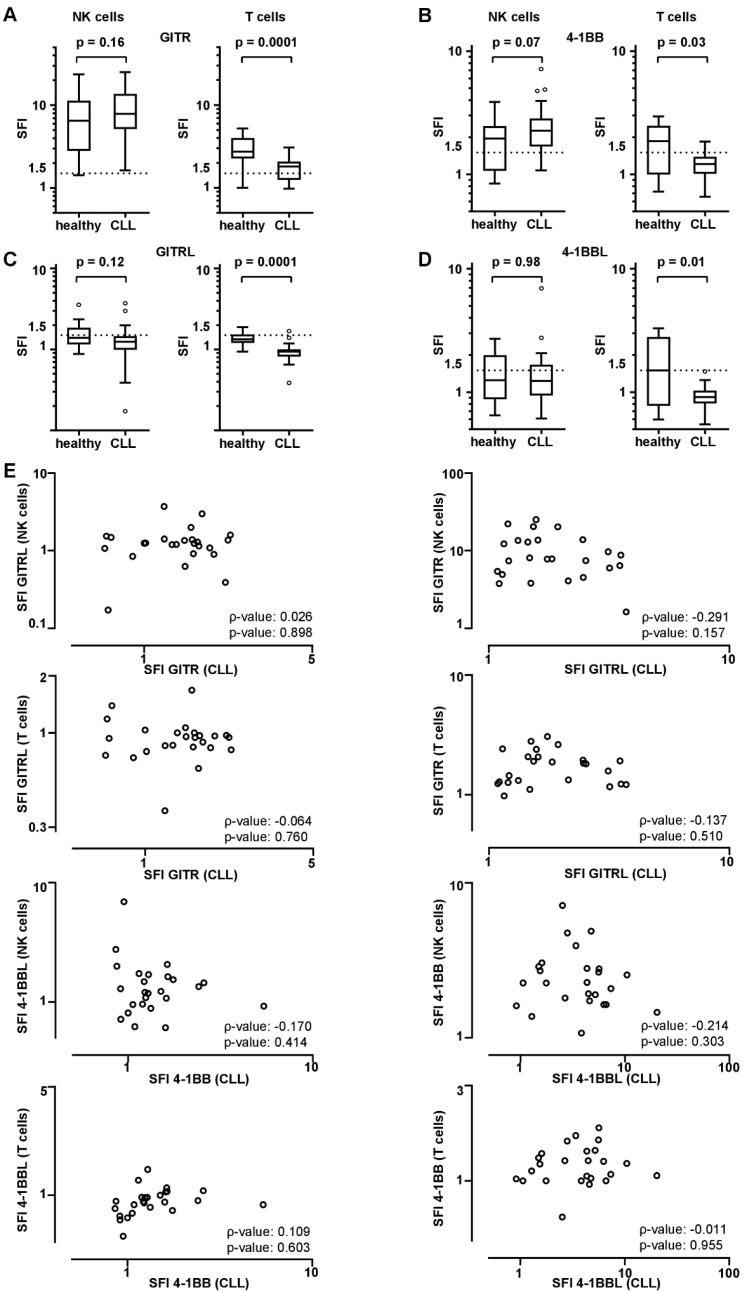
GITR/GITRL and 4-1BB/4-1BBL expression on peripheral NK and T cells of CLL patients and healthy individuals. GITR/GITRL/4-1BB/4-1BBL expression levels were analyzed on NK and T cells by flow cytometry. (**A**–**D**) GITR/GITRL/4-1BB/4-1BBL expression levels on peripheral NK and T cells of CLL patients (*n* = 25) and healthy donors (*n* = 15) are demonstrated as SFI levels of GITR, GITRL (**A**,**C**), 4-1BB, and 4-1BBL (**B**,**D**) positive samples (boxplots with min/max whiskers). (**E**) Correlation of GITR and GITRL, and 4-1BB and 4-1BBL SFI levels on CLL cells vs. NK or T cells is shown (single values, spearman correlation (ρ)). (**A**–**D**) Statistical analysis with Mann-Whitney-U test.

NK cells of CLL patients were shown to have GITR, GITRL, 4-1BB, and 4-1BBL expression levels up to SFIs of 25.2, 3.7, 7.1, and 6.9, whereas T cells of the same patients expressed SFIs up to 3.1, 1.7, 1.8, and 1.5, respectively (Figure 2A–D). Expression level on NK and T cells of healthy PBMCs was analyzed, and expression levels on NK cells were up to 23.7, 3.5, 3.8, and 2.4, whereas T cells revealed expression levels up to 4.3, 1.8, 2.9, and 3.2 for GITR, GITRL, 4-1BB, and 4-1BBL, respectively (Figure 2A–D). Although expression of GITR, GITRL, 4-1BB, and 4-1BBL was comparable on NK cells of CLL patients and healthy individuals, T cells of healthy volunteers expressed higher levels of GITR, GITRL, 4-1BB, and 4-1BBL compared to CLL patients’ T cells. Correlation of GITR and GITRL or 4-1BB and 4-1BBL expression on CLL versus NK or T cells did not show a significant correlation, as shown in Figure 2E. 

**Figure 3:**
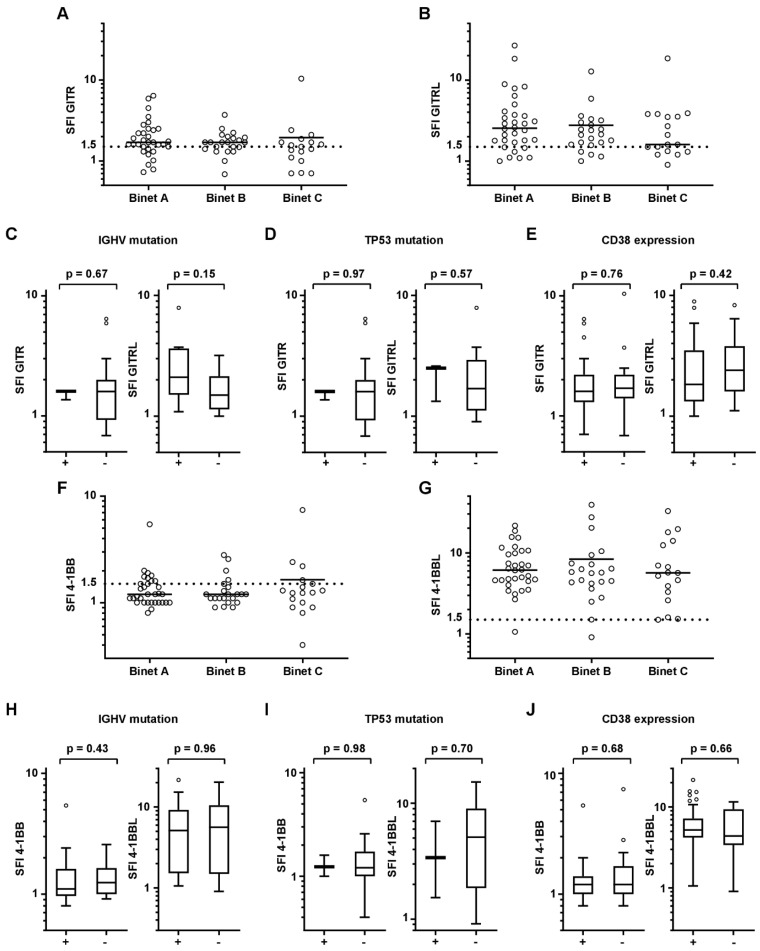
GITR, GITRL, 4-1BB, or 4-1BBL expression of CLL cells are classified by clinical characteristic. Binet stages, IGHV mutation, TP53 mutation, and CD38 positivity of CLL cells classified for each GITR, GITRL, 4-1BB, and 4-1BBL expression (SFI). (**A**,**B**,**F**,**G**) Distribution of GITR/GITRL/4-1BB/4-1BBL expression (SFI) according to Binet stage at sample acquisition is shown (single values, mean). (**C**,**H**) IGHV mutation status (− unmutated, + mutated). (**D**,**I**) TP53 mutation status (unmutated, + mutated). (**E**,**J**) CD38 expression (>30%) of CLL cells are demonstrated for GITR/GITRL/4-1BB/4-1BBL expression (SFI). (**C**–**E**,**H**–**J**) Statistical analysis with Mann-Whitney-U test.

Furthermore, no significant difference was noted for GITR, GITRL, 4-1BB, and 4-1BBL levels according to different Binet stages at the time of sample acquisition (Figure 3A,B,F,G). A tendency for higher GITR expression was observed for Binet C vs. Binet A, but failed to reach statistical significance (*p* = 0.070). Patients were then grouped for IGHV mutational status, TP53 mutation, and CD38 positivity. Although FISH and molecular genetics data are not available for all patients, no difference was observed for GITR, GITRL, 4-1BB, and 4-1BBL between the different groups (Figure 3C–E,H–J).

### 3.3. 4-1BB Expression Is Prognostic of Outcome in CLL

**Figure 4:**
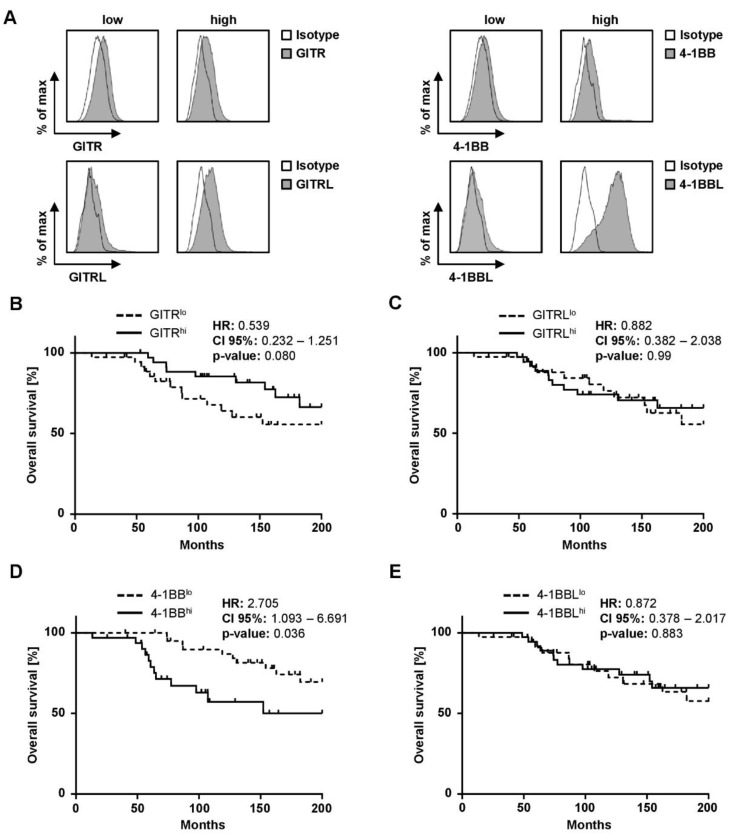
Impact of GITR, GITRL, 4-1BB, or 4-1BBL expression on CLL patients’ survival. (**A**) Exemplary histogram plots for each surface marker of high and low expressing patients are shown by flow cytometry. (**B**–**E**) Overall survival of CLL patients according to GITR^lo^ and GITR^hi^, GITRL^lo^ and GITRL^hi^, 4-1BB^lo^ and 4-1BB^hi^, and 4-1BBL^lo^ and 4-1BBL^hi^ expression (SFI) in Kaplan-Meier analysis. Low and high GITR/GITRL/4-1BB/4-1BBL expression levels are sub-grouped according to median of SFI levels of surface markers. Median OS for GITR^hi^ (continuous line) and GITR^lo^ cases (dotted line) was not reached (log-rank test). Median OS was reached in GITRL^hi^ (continuous line) and GITRL^lo^ (dotted line) was not reached (log-rank test). In 41BB^hi^ the median OS was 152 month (continuous line) and was not be reached in 4-1BB^lo^ (dotted line) and differed significantly (log-rank test). Median OS in 4-1BBL^hi^ and 4 1BBL^lo^ was not reached (dotted line; log-rank test).

Patients were divided into two groups (high (>median SFI) and low (≤median SFI)) by using SFI expression profile of each marker. Figure 4A shows exemplary histogram plots for each surface marker of high and low expressing cells of patients. In order to further explore the impact of GITR/GITRL and 4-1BB/4-1BBL expression on survival in CLL and to evaluate the prognostic value of our selected cut-off, Kaplan-Meier analyses were performed. When grouped according to GITR expression, GITR^hi^ cases had a tendency to longer OS (Figure 4B, *p* = 0.080). Median OS in GITR^hi^ and GITR^lo^ cases was not reached. In contrast, expression levels of GITRL did not at all correlate with OS (*p* = 0.987) (Figure 4C). 

When grouped according to 4-1BB expression, 4-1BB^hi^ cases exhibited a significantly shorter OS (Figure 4D, *p* = 0.036). Median OS in 4-1BB^hi^ cases was 152 months, whereas median OS in 4-1BB^lo^ was not reached. Of note, the expression levels of the respective ligand, 4-1BBL, alike with the GITR molecule system, did not correlate with OS (Figure 4E, *p* = 0.883).

## 4. Discussion

The development and clonal evolution of malignant cells to clinically apparent disease is dependent on their ability to survive and evade immune surveillance [34,35] as, among others, expression of the immunoreceptors GITR and 4-1BB and their ligands determines whether malignant cells are eliminated by immune effector cells [25,33]. In turn, expression of these receptors and their ligands might accordingly influence disease outcome. Here, we report on the prognostic relevance of GITR/GITRL and 4-1BB/4-1BBL for OS in CLL.

Using flow cytometric analysis, we found GITR expression on CLL cells in 65.7% of patients, whereas expression of GITR ligand was higher (73.9%). There was no positive correlation between GITR and GITRL expression, indicating that a relevant co-expression on CLL cells might be rare. 4-1BB was found to be expressed in our CLL cohort in a relevant proportion (27.4%) of CLL cells. Similar to GITR and GITRL, expression of 4-1BBL was higher than that of 4-1BB (94.5% vs. 27.4%), and a relevant co-expression of receptor and ligand was not observed. The results on the expression of both molecule systems are in line with other previous publications [21,25,33,36].

In addition, our analyses of GITR/GITRL and 4-1BB/4-1BBL expression on CLL patients’ NK and T cells revealed pronounced expression of GITR and 4-1BB on NK cells of CLL patients, whereas GITRL and 4-1BBL were only very weakly expressed. Expression of GITR/GITRL and 4-1BB/4-1BBL on NK cells of healthy donors was comparable to CLL patients. In contrast, T cells showed higher expression of GITR/GITRL and 4-1BB/4-1BBL in healthy controls compared to CLL patients. This might point to an immunosuppressive microenvironment in CLL patients. No correlations of GITR/GITRL and 4-1BB/4-1BBL expression on CLL cells and patients’ NK and T cells was observed, which may point to a subordinate role of NK or T cell interactions with CLL cells in regard to these receptor-ligand systems. 

As the therapeutic landscape of CLL has changed in the past years, new prognostic markers are highly needed, especially as the disease still remains a permanent companion. By introducing the CLL-IPI score in 2018, physicians were provided with treatment guidance [9]. However, the CLL-IPI score requires assessment of genetic markers, which are costly to determine and change over time [37,38]. This is also represented in our cohort of CLL patients, as IGHV mutational status was only available for 23% of patients. Flow cytometry-assessed prognostic markers such as CD38 and ZAP70 were not included in the training data set of CLL-IPI score [38]. Of note, diagnosis of CLL is still based on immunophenotyping of peripheral lymphocytes [39]. Thus, establishing prognostic flow cytometric markers that can be analyzed simultaneously is of special interest as described for other markers and entities [40,41], as it would be efficient with regard to time and costs. Whereas 4-1BB expression seemed to be higher in Binet C patients, all other prognostic markers showed no correlation with the 4-1BB/4-1BBL and GITR/GITRL expression profile. It should be noted that available data on established prognostic markers were limited in our cohort.

Buechele et al. identified inhibition of NK cell activity by GITRL on CLL cells. NK reactivity is essential for induction of antibody dependent cellular cytotoxicity (ADCC) and thus efficacy of anti-CD20 antibody treatment [25]. Using the median expression levels of GITR or GITRL as a cut-off, GITR trended towards a correlation with OS in CLL, indicating a prognostic role in CLL as reported for platelet derived GITR in breast cancer [42]. Of note, a difference in OS was not observed for GITRL. In addition to the possible impact on survival, several clinical trials investigate GITR as therapeutic target using an anti-GITR antibody (NCT01239134, NCT02598960), which, based on our data, might be a promising approach for GITR^+^ CLL as well.

In T cells, the role of 4-1BB has been extensively studied, and activation of 4-1BB leads to an increased production of IL-2 by effector T cells, mirrored by enhanced proliferation and cytotoxicity [43]. In addition, 4-1BB signaling influences other compartments of the cellular immune defense like B cells by induction of proliferation and immunoglobulin production [44]. Downstream signaling of 4-1BB involves NF-κB, which is a main target of B cell receptor (BCR) activation [45]. It was shown that induction of 4-1BB on CLL B cells have an effect on the prevention of apoptosis by activating NF-κB signaling [46]. Since our data showed that 4-1BBL expression on CLL cells is higher compared to patients’ NK or T cells, auto-stimulation of CLL cells by the 4-1BB/4-1BBL-axis might be one reasonable explanation for the worse OS. Inhibitors of the BCR signaling pathway like ibrutinib or acalabrutinib are effective in treatment of CLL and other hematologic neoplasia, underlining the relevance of the downstream process of 4-1BB. This is supported by our finding that patients with high 4-1BB expression on CLL B cells have a shorter OS. This is in contrast to data on 4-1BB on AML blasts [47] and might be explained by the different disease biology.

Choi et al. demonstrated that 4-1BB signaling of myeloid cells negatively regulates peripheral T cells [48]. It is tempting to speculate that CLL cells might likewise modify the environment and T cell recognition via the 4-1BB/4-1BBL axis. In NK cells, available data on the role and function of 4-1BB are at least partially conflicting [49,50]. Baessler et al. reported that activation of 4-1BB in mice leads to activation of NK cells with an amplification of the cytotoxic response, whereas in humans NK cell response is suppressed [51]. The increased expression of 4-1BBL may cause a decreased natural and rituximab-induced response (cytolysis, ADCC) of NK cells against CLL cells. A blockade of 4-1BB in cultures with CLL and NK cells led in reverse to an increase in granule mobilization, perforin release, cytotoxicity, and interferon-γ production of the NK cells [33]. Further studies are needed to elucidate the role of 4-1BB/4-1BBL and GITR/GITRL expression in the context of NK cell reactivity. 

## 5. Conclusions

In conclusion, to our knowledge this is the first analysis of the prognostic role of 4-1BB/4-1BBL and GITR/GITRL surface levels on CLL cells in a patient cohort with long observational time. We introduce a flow cytometric approach that can be implemented at time of diagnosis and may overcome the limitations associated with the use of genetic markers. The observed association of 4-1BB expression with OS may thus serve as easily available prognostic marker in CLL.

## Figures and Tables

**Table 1:** Patient characteristics.

	Number of Patients (%) (Total Number of Patients *n* = 73)
Sex	
Male	42 (58)
Female	31 (42)
Median age at diagnosis (years)	63 (range 36–80)
Binet stage initial diagnosis	
A	48 (66)
B	15 (21)
C	6 (8)
not available	4 (5)
Rai stage initial diagnosis	
0	20 (27)
I–II	32 (44)
III–IV	6 (8)
not available	15 (21)
Binet stage sample acquisition	
A	32 (44)
B	22 (30)
C	17 (23)
not available	2 (3)
Rai stage sample acquisition	
0	12 (16)
I–II	38 (53)
III–IV	21 (28)
not available	2 (3)
IGHV mutational status	
mutated	10 (59)
unmutated	7 (41)
CD38 expression	
<20%	43 (59)
20–29%	3 (4)
≥30%	15 (21)
not available	12 (16)
Cytogenetics risk	
favorable *	5 (7)
intermediate *	4 (5)
poor *	10 (14)
not available	54 (74)
TP53 mutation	
Positive	3 (4)
negative	20 (27)
not available	50 (68)
Lymphocyte count (1/µL)	43,984 (range 5366–320,330)
Hb (g/dL)	12.7 (range 7.9–16.8)
Plt (1000/µL)	183 (range 14–346)
β-2 microglobuline (mg/L)	3.7 (range 1.7–9.7)

Hb: haemoglobin; plt: thrombocytes; * classical cytogenetic analyses were performed, and patient grouped accordingly: favorable: 12q trisomy, del 13q; intermediate: normal karyotype; poor: del17p, del11q.

## Data Availability

The data presented in this study are available on request from the corresponding author. The data are not publicly available due to the used patient material.
